# *RNF213*-Related Vasculopathy: An Entity with Diverse Phenotypic Expressions

**DOI:** 10.3390/genes16080939

**Published:** 2025-08-07

**Authors:** Takeshi Yoshimoto, Sho Okune, Shun Tanaka, Hiroshi Yamagami, Yuji Matsumaru

**Affiliations:** 1 Department of Stroke and Cerebrovascular Diseases, University of Tsukuba Hospital, Tsukuba 305-8576, Japan; sho.oku27@md.tsukuba.ac.jp (S.O.); tanaka.shun.ge@ms.hosp.tsukuba.ac.jp (S.T.); yamagami.hiroshi@md.tsukuba.ac.jp (H.Y.); yujimatsumaru@md.tsukuba.ac.jp (Y.M.); 2 Division of Stroke Prevention and Treatment, Institute of Medicine, University of Tsukuba, Tsukuba 305-8576, Japan; 3 Department of Neurosurgery, Institute of Medicine, University of Tsukuba, Tsukuba 305-8576, Japan

**Keywords:** stroke genetics, *RNF213*-related vasculopathy, moyamoya disease

## Abstract

Moyamoya disease (MMD) is primarily associated with genetic variants in *RNF213*. *RNF213* p.R4810K (c.14429G>A, p.Arg4810Lys) is a founder variant predominantly found in East Asian populations and is strongly associated with MMD, a rare cerebrovascular condition characterized by progressive stenosis of intracranial arteries and the development of abnormal collateral networks. Recent evidence suggests that *RNF213* variants are also enriched in non-moyamoya intracranial arteriopathies, such as large-artery atherosclerotic stroke and intracranial arterial stenosis/occlusion (ICASO), particularly in east Asian individuals with early-onset or cryptogenic stroke. This expanded phenotypic spectrum, termed *RNF213*-related vasculopathy (RRV), represents a distinct pathogenic entity that may involve unique pathogenic processes separate from traditional atherosclerosis. In this review, we synthesize current genetic, clinical, radiological, and experimental findings that delineate the unique features of RRV. Patients with RRV typically exhibit a lower burden of traditional vascular risk factors, negative vascular remodeling in the absence of atheromatous plaques, and an increased propensity for disease progression. *RNF213* variants may compromise vascular resilience by impairing adaptive responses to hemodynamic stress. Furthermore, emerging cellular and animal model data indicate that *RNF213* influences angiogenesis, lipid metabolism, and stress responses, offering mechanistic insights into its role in maintaining vascular integrity. Recognizing RRV as a distinct clinical entity has important implications for diagnosis, risk stratification, and the development of genome-informed therapeutic strategies.

## 1. Introduction

Moyamoya disease (MMD) is a rare idiopathic cerebrovascular disorder characterized by progressive steno-occlusion at the terminal portions of the intracranial internal carotid arteries, accompanied by the formation of fragile collateral vessels known as “moyamoya vessels” [1,2,3,4]. A major breakthrough in elucidating MMD’s genetic basis occurred in 2011, when genome-wide association studies in East Asian populations identified ring finger protein 213 (*RNF213*), also known as mysterin, as the first susceptibility gene [5,6,7]. The p.R4810K (c.14429G>A, rs112735431) variant in *RNF213* demonstrates remarkable ethnic specificity: it is found in approximately 80–90% of familial MMD cases in Japan and Korea yet is virtually absent in Caucasian populations [6,7,8,9,10,11]. This ethnic distribution parallels the geographic prevalence of MMD itself, with incidence rates of 0.5–1 per 100,000 in East Asia compared to substantially lower rates in Western countries [12,13,14]. Despite its strong association with MMD, *RNF213* p.R4810K exhibits low penetrance, with only approximately 1 in 150 carriers developing overt disease. This incomplete penetrance suggests that additional environmental or genetic “second-hit” factors are required to modulate clinical expression [6,7,15]. In the following sections, we systematically examine the cerebrovascular phenotype, risk factor profile, natural history, and clinical outcomes of *RNF213*-related vasculopathy (RRV), synthesizing insights from advanced neuroimaging studies, prospective human cohorts, and mechanistic data from emerging experimental models.

## 2. Systemic Associations of RRV

### 2.1. Intracranial Arterial Stenosis/Occlusion

Recent evidence has expanded the phenotypic spectrum of *RNF213* p.R4810K beyond classical MMD to encompass a broader range of intracranial large-artery diseases [1,4,16,17,18]. Carriers may develop intracranial arterial stenosis/occlusion (ICASO) that does not fulfill the angiographic criteria for MMD and has frequently been misclassified as large-artery atherosclerosis (LAA) [16,17,18,19]. A landmark case–control study of 46,958 Japanese individuals (17,752 stroke patients and 29,206 controls) provided compelling evidence for this expanded phenotype: *RNF213* p.R4810K nearly doubled the overall ischemic stroke risk [odds ratio (OR) ~1.9] and increased LAA stroke risk by more than threefold. Notably, the effect was most pronounced in females, and carriers experienced strokes at a significantly younger age (on average, 4 years earlier than non-carriers) [16]. These findings have catalyzed the emergence of “*RNF213*-related vasculopathy” as a unifying concept [1,4], which encompasses a clinical continuum from asymptomatic carriers through ICASO and ischemic stroke (IS)/transient ischemic attack (TIA) to full-blown MMD. In this conceptual framework, *RNF213* p.R4810K underlies a shared arteriopathy that manifests with variable severity—from mild stenosis to bilateral occlusive disease with characteristic moyamoya collaterals [13,16,19]. Supporting evidence comes from clinical observations within MMD families, where relatives carrying p.R4810K may present with ICASO or conventional IS patterns rather than classical MMD [16,18]. This phenotypic heterogeneity suggests that RRV represents a distinct genetic endophenotype that blurs traditional diagnostic boundaries between MMD and non-MMD intracranial steno-occlusive disease. The recognition of RRV has important diagnostic and therapeutic implications. Some investigators have proposed reclassifying ischemic strokes in *RNF213* variant carriers—particularly those lacking overt moyamoya vessels—as “stroke of other determined etiology (non-inflammatory arteriopathy)” rather than conventional atherosclerosis under the TOAST classification system [13,16,19]. This reclassification acknowledges the distinct pathophysiology underlying RRV and may guide more targeted therapeutic approaches.

### 2.2. Systemic Hypertension

Recent genetic and epidemiological studies have consistently demonstrated that the *RNF213* p.R4810K variant is associated with increased susceptibility to systemic hypertension. A prospective cohort study of 9153 Japanese adults without prior cardiovascular disease found that carriers of the *RNF213* p.R4810K variant had a significantly higher incidence of cardiovascular events (71.0 vs. 26.9 per 10,000 person-years, *p* = 0.009) over an 8.5-year follow-up period. The variant was independently associated with increased systolic blood pressure (BP) (coefficient, 8.19 mmHg; *p* < 0.001) and remained an independent risk factor for cardiovascular disease and major cardiac events, even after adjustment for confounding factors. However, its association with total stroke was not statistically significant. These findings suggest that the *RNF213* variant increases both BP and cardiovascular risk in the general Japanese population, underscoring its significance beyond MMD [20]. Moreover, population-based studies have shown that carriers of the p.R4810K allele exhibit a significant elevation in systolic BP, with reported increases ranging from 8 to 19 mmHg compared to non-carriers, independent of overt major vascular disease [21]. This effect is observed even in individuals without clinical or radiological signs of cerebrovascular pathology, suggesting a fundamental role for *RNF213* in regulating systemic vascular tone. Mechanistically, altered function of vascular smooth muscle cells (VSMCs) and endothelial cells driven by the *RNF213* variant may disrupt homeostatic signaling pathways, notably those involving nitric oxide synthesis, vascular remodeling, and inflammatory responses. These pathways are critical for maintaining BP and vascular reactivity, and their perturbation may underlie the hypertension phenotype observed in carriers.

Several studies have reported increased pulse wave velocity and decreased arterial compliance in carriers of the *RNF213* variant, even in the absence of overt vascular disease. These findings suggest a subclinical increase in arterial stiffness, potentially mediated by smooth muscle dysfunction and aberrant extracellular matrix remodeling, consistent with the effects of *RNF213* dysfunction and impaired caveolin-1 signaling [6].

### 2.3. Early-Onset Coronary Artery Disease and Myocardial Infarction

Building upon the association with cerebrovascular pathology, the *RNF213* p.R4810K variant has also been robustly linked to early-onset coronary artery disease (CAD) and myocardial infarction (MI). Multiple case–control studies from Japan and East Asia report that carriers of the variant have a 2.9–3.8-fold increased risk for premature CAD and MI, a risk that remains significant even after multivariate adjustment for conventional risk factors such as hypertension or dyslipidemia [22]. Notably, this increased risk is observed in both symptomatic patients and, in certain datasets, asymptomatic individuals, highlighting the inherited predisposition conferred by the *RNF213* locus. The effect seems especially pronounced in younger individuals and in specific at-risk subgroups, such as females or those with metabolic risk profiles. A unique feature of *RNF213*-related CAD risk is its apparent independence from typical patterns of atherosclerotic plaque burden or traditional cardiovascular risk factors. Rather, it may reflect an underlying predisposition to non-atherosclerotic coronary vasculopathy, microvascular disease, or abnormal arterial remodeling, as further discussed below.

### 2.4. Coronary Vasospasm: Vasospastic Angina

A large-scale Japanese case-control study including 1713 individuals with vasospastic angina (VSA) and 3347 controls found that carriers of the *RNF213* p.R4810K variant had an approximately 3.7-fold increased risk of developing VSA (adjusted odds ratio [OR] 3.73, 95% CI 2.63–5.29; *p* < 0.001). Subgroup analyses revealed that the risk was even higher in female carriers (OR 6.04) and those with dyslipidemia (OR 6.03), suggesting the presence of gene–environment interactions analogous to those proposed in MMD [23].

### 2.5. Pulmonary Arterial Hypertension

Beyond its established role in cerebrovascular and coronary pathology, recent investigations have highlighted a significant involvement of *RNF213*, particularly the p.R4810K variant, in pulmonary vascular disease. Notably, the frequency of this variant is markedly increased in patients with pulmonary arterial hypertension (PAH), both in Japanese and Korean cohorts, and there is accumulating evidence of homozygous carriers manifesting with severe, early-onset PAH [24]. *RNF213*, a susceptibility gene for MMD, has also been recently implicated in extracranial vascular diseases, such as pulmonary hypertension (PH). In this study, genetic screening of 27 Japanese patients with PH identified two rare, MMD-associated *RNF213* variants (p.R4810K and p.A4399T) in two patients; three BMPR2 mutations were also found, but no CAV1 mutations. Functional experiments in mice showed that overexpression of an endothelial cell-specific *RNF213* mutant, but not wild-type or ablated *RNF213*, worsened hypoxia-induced PH phenotypes, including elevated right ventricular pressure, hypertrophy, and increased muscularization of pulmonary vessels. Electron microscopy revealed endothelial cell detachment, and Western blotting showed a significant reduction in caveolin-1 in the lungs of mutant mice, indicating endothelial dysfunction. These results suggest that *RNF213* is a genetic risk factor for PH and may contribute to systemic vasculopathy [25]. This suggests a striking dose-dependent effect of *RNF213* dysfunction on the pulmonary arterial system. Direct associations between *RNF213* p.R4810K homozygosity and more severe, early-onset (juvenile) and aggressive forms of PAH or multifocal pulmonary vascular lesions have been repeatedly documented. In several genetic and clinical studies, individuals who are homozygous (carry two copies) for p.R4810K display a markedly higher risk for earlier disease onset, greater severity, and involvement of multiple vascular territories—including pulmonary arteries, cerebral arteries, and sometimes even systemic arteries—compared to heterozygous or wild-type individuals [26,27]. Cases of aggressive juvenile PAH, often with multifocal or systemic vasculopathy (sometimes termed “systemic vascular diseases”), have been directly linked to this genotype, underscoring a gene-dosage effect that is far more pronounced than in carriers with only one affected allele [28].

Moreover, a Japanese multicenter prospective cohort study investigated the relationship between the *RNF213* p.R4810K variant and echocardiographic findings in Japanese patients with cerebrovascular diseases, focusing on preclinical cardiovascular changes [29]. Out of 2089 patients genotyped, 71 *RNF213* p.R4810K carriers and 1241 non-carriers without chronic heart or pulmonary diseases were analyzed. Carriers of the p.R4810K variant had a significantly longer right ventricular outflow tract acceleration time (RVOT-ACT) in multivariable linear regression (β = 8.33 ms, 95% CI: 0.92–15.74, *p* = 0.028) and displayed higher odds of having RVOT-ACT ≥ 150 ms (odds ratio, 2.22; 95% CI: 1.18–4.18; *p* = 0.014) compared to non-carriers. The study concludes that longer RVOT-ACT in variant carriers may reflect expansion of the pulmonary vascular bed due to abnormal collateral networks and capillary dilation in the early, preclinical stage of *RNF213*-related pulmonary hypertension. The results underscore the need for careful multi-organ evaluation in individuals with cerebrovascular diseases who carry this variant, as RRV extends beyond the brain to involve systemic cardiovascular disease.

The clinical spectrum in *RNF213* variant carriers is notably broad. Some individuals present with isolated PAH, while others may show a combination of pulmonary hypertension, systemic arteriopathy (renal/visceral involvement), or cerebral large-vessel disease—sometimes within the same family. In many cases, the onset of PAH in variant carriers occurs at a much younger age and follows a more aggressive trajectory than in idiopathic or *BMPR2*-mutant forms [30,31,32]. Patients may demonstrate resistance to standard pulmonary vasodilator therapy, and vascular imaging often reveals evidence of segmental stenoses or occlusions, reminiscent of the steno-occlusive changes seen in the brain.

### 2.6. Sporadic Aortic Dissection

Recent large-scale Chinese cohort studies and reviews have demonstrated a clear association between the *RNF213* gene and sporadic aortic dissection. Specifically, in a panel sequencing study of 702 patients with sporadic aortic dissection, pathogenic or likely pathogenic *RNF213* variants were identified in 3.7% (26/702) of patients. Notably, among these, 7 patients carried *RNF213* variants despite having no abnormalities in well-known aortic dissection risk genes such as FBN1, ACTA2, or MYH11. This suggests that *RNF213* mutations can contribute to disease onset independently of the traditional risk genes [33]. Furthermore, transcriptome analysis has shown a correlation between *RNF213* mRNA expression and that of FBN1 (the gene most frequently implicated in aortic dissection). Reduced *RNF213* expression may be involved in abnormal aortic development or increased aortic fragility, indicating a possible mechanistic role for *RNF213* deficiency in disease pathogenesis.

### 2.7. Carotid Artery

A large-scale registry study in Japan investigated the association between the *RNF213* p.R4810K variant and the outer diameter of cervical arteries—including the common carotid artery (CCA), internal carotid artery (ICA), and vertebral artery (VA)—in 617 Japanese patients with IS, using carotid ultrasonography [34]. Among the cohort, 4.2% (26 patients) were identified as carriers of the p.R4810K variant. Variant carriers also had significantly smaller outer arterial diameters: CCA (7.25 mm vs. 8.22 mm; adjusted OR per 1 mm decrease = 2.94; 95% confidence interval [CI], 1.69–5.00; *p* < 0.01), cervical ICA (4.99 mm vs. 5.55 mm; adjusted OR = 1.66; 95% CI, 1.03–2.70; *p* = 0.03), and cervical VA (3.55 mm vs. 4.10 mm; adjusted OR = 2.56; 95% CI, 1.33–4.76; *p* < 0.01). Sensitivity analyses restricted to patients without distal vascular stenosis or occlusion revealed consistent findings, with significantly reduced diameters observed in CCA (adjusted OR = 3.44), ICA (adjusted OR = 2.04), and VA (adjusted OR = 3.23) among variant carriers.

### 2.8. Stenosis of Abdominal Branches of the Aorta

In a prospective screening study conducted by Jee et al., extracranial systemic arteriopathy was evaluated in 63 young adults (aged <50 years) diagnosed with MMD. The researchers used contrast-enhanced magnetic resonance angiography to assess systemic arteries outside the cranium, including the aorta, renal, celiac, mesenteric, and iliac arteries. Extracranial systemic artery stenosis was identified in 25.4% of participants (16 of 63), with renal artery involvement being the most frequent (14.3%), followed by stenoses in the celiac (9.5%) and iliac (4.8%) arteries. Notably, the prevalence of systemic arteriopathy was significantly higher in patients carrying the *RNF213* p.R4810K variant compared to non-carriers (33.3% vs. 0.0%, *p* = 0.01) [35].

### 2.9. Neurocristopathy and Rare Syndromes

Rare *RNF213* variants have also been identified in PHACE syndrome—a complex neurocristopathy characterized by infantile hemangiomas and diverse vascular malformations—as well as in cases of intracranial arterial dissection. These observations extend the clinical spectrum of *RNF213* beyond adult-onset steno-occlusive disorders to include congenital and pediatric vascular anomalies, highlighting its fundamental role in early vascular patterning and arterial wall integrity. The association of *RNF213* mutations with PHACE syndrome, which features a wide range of arterial anomalies affecting the aortic arch, cerebral vasculature, and cardiovascular structures, underscores the gene’s broad developmental influence [36,37]. Further emphasizing its developmental and systemic significance, a cerebrovascular imaging study in non-moyamoya patients with IS or TIA demonstrated that carriers of the *RNF213* p.R4810K variant exhibit distinctive configurations of the circle of Willis. Specifically, these carriers were markedly more likely than non-carriers to have bilateral posterior communicating arteries (56% vs. 13%, *p* < 0.01) and, conversely, less likely to have an anterior communicating artery (68% vs. 84%, *p* = 0.04). These associations remained robust after multivariable adjustment, supporting the idea that *RNF213* influences genetically determined, developmentally rooted arterial remodeling in the cerebral circulation [38].

**Figure 1:**
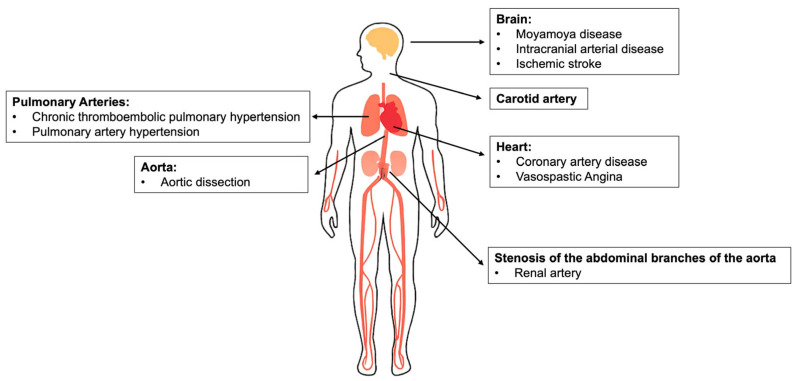
Systemic vascular manifestations associated with the *RNF213* p.R4810K variant.

Systemic vascular manifestations associated with the *RNF213* p.R4810K variant are shown in Figure 1.

This schematic illustrates the broad spectrum of vascular diseases associated with the east Asian–specific *RNF213* p.R4810K variant. The affected vascular territories span multiple organ systems, including:

- Brain: Moyamoya disease, intracranial arterial stenosis, and ischemic stroke
- Carotid artery: Large-artery atherosclerosis or non-atherosclerotic narrowing
- Heart: Coronary artery disease and vasospastic angina
- Pulmonary arteries: Chronic thromboembolic pulmonary hypertension and pulmonary arterial hypertension
- Aorta: Aortic dissection
- Abdominal aortic branches: Renal artery stenosis

These phenotypes reflect the systemic nature of *RNF213*-related vasculopathy beyond the cerebral vasculature. The diagram underscores the importance of comprehensive vascular screening and interdisciplinary management in variant carriers.

## 3. Molecular Mechanisms

### 3.1. RNF213 Structure and Enzymatic Functions

*RNF213*, also known as mysterin, is an exceptionally large protein composed of 5207 amino acids with an approximate molecular weight of 591 kDa, placing it among the largest proteins in the human proteome [5,6,39]. It contains two tandem AAA+ ATPase domains that confer mechanochemical activity, and a C-terminal RING finger domain that characterizes E3 ubiquitin ligases. *RNF213* exhibits E3 ligase activity, with at least two identified substrates: nuclear factor of activated T-cells 1 (NFAT1) and filamin A [40,41,42]. Through targeted ubiquitination, *RNF213* regulates essential endothelial signaling pathways, including the non-canonical Wnt/Ca^2+^–NFAT axis that modulates endothelial nitric oxide synthase, vascular tone, and angiogenic gene expression [7,40,41,42,43]. It promotes vascular stability by facilitating NFAT1 degradation and regulating filamin A, thereby curbing aberrant angiogenesis. Loss of *RNF213* impairs vessel pruning, resulting in the persistence of tortuous, ineffective collaterals reminiscent of those found in moyamoya vessels. Conversely, excessive *RNF213* activity may inhibit collateral formation, underscoring the necessity for a finely balanced regulatory mechanism [6,41].

### 3.2. Impact of the p.R4810K Mutation

The east Asian–specific *RNF213* p.R4810K (Arg4810Lys) variant resides within the second AAA+ domain and alters the enzymatic characteristics of the protein [5,6,41]. Biochemical studies have shown that this mutation reduces ATPase activity and affects oligomerization, disrupting *RNF213*’s normal function [44]. In endothelial cells, wild-type *RNF213* requires interferon-β stimulation to exert its antiangiogenic effects, whereas the R4810K variant exerts these effects constitutively, reflecting a gain-of-function or hypermorphic phenotype [45,46]. This may stem from enhanced stability of oligomeric conformations that prolong degradation signals for NFAT1 and filamin A [47,48]. Supporting this, endothelial-specific transgenic mice expressing the R4757K ortholog exhibit impaired neovascularization under hypoxic conditions, consistent with defective collateral formation in variant carriers [39,41,42,45,46].

### 3.3. Endothelial Function and Inflammation

*RNF213* has emerged as a key regulator of endothelial homeostasis and blood–brain barrier (BBB) integrity [46,47,48,49]. Knockout studies in human brain endothelial cells reveal increased permeability, abnormal morphology, and features consistent with endothelial activation and BBB disruption [49]. *RNF213*-deficient cells exhibit enhanced proliferation, migration, and tube formation, indicating a shift toward a hyperplastic, proangiogenic phenotype [41,42,48]. Mechanistically, *RNF213* knockdown activates angiogenic pathways such as Hippo–YAP, driving maladaptive vascular responses. As *RNF213* is upregulated by interferons, it may link inflammatory stimuli to vascular remodeling [44,50,51]. Through its E3 ligase activity, *RNF213* also influences other immune-related pathways, positioning it at the intersection of angiogenesis, vascular barrier function, and immune regulation [41,51,52].

### 3.4. Caveolin-1

Recent studies have identified caveolin-1 (Cav-1)—a membrane scaffolding protein critical for caveolae formation, nitric oxide signaling, and vascular tone—as a direct *RNF213* substrate [53]. *RNF213* binds Cav-1 via its catalytically active AAA+ A3 domain in an ATP-dependent manner and facilitates Cav-1 polyubiquitination at four N-terminal lysines (K26, K47, K57, K65) [53,54]. It also regulates Cav-1 phosphorylation at Tyr14, a key site controlling nitric oxide (NO) bioavailability and endothelial responses to oxidative stress. Both disease-associated RING domain mutations (e.g., C3997Y) and the p.R4810K variant impair *RNF213*’s E3 ligase activity toward Cav-1 while preserving physical interaction, suggesting dysfunctional Cav-1 signaling as a pathogenic mechanism. In vitro, *RNF213* knockdown enhances Cav-1 Tyr14 phosphorylation and increases NO production under oxidative conditions, supporting *RNF213*’s role as a brake on endothelial hyperactivation [53].

Beyond its function as a structural component of caveolae and regulator of nitric oxide signaling, Cav-1 is increasingly recognized as a key player in the pathogenesis of arterial stiffness, a major cardiovascular disease risk factor. Reduced or dysfunctional Cav-1 expression correlates with heightened vascular stiffness in multiple pathological contexts, including chronic kidney disease, dyslipidemia, aging, and PAH [55,56]. Mechanistically, Cav-1 modulates vascular smooth muscle cell (VSMC) contractility, facilitates low-density lipoprotein (LDL) transcytosis across the endothelium, regulates pro-inflammatory signaling cascades, and orchestrates extracellular matrix (ECM) remodeling, all vital for maintaining arterial compliance and integrity [55,57,58].

In the context of RRV, impaired Cav-1 ubiquitination and phosphorylation resulting from *RNF213* mutations may cause aberrant endothelial and VSMC responses, thereby promoting maladaptive vascular remodeling and arterial stiffening [55]. Given these multifaceted roles, Cav-1 represents a promising therapeutic target for RRV, meriting thorough exploration in both preclinical and clinical settings.

### 3.5. Lipid Metabolism

Beyond vascular signaling, *RNF213* also regulates lipid metabolism via its association with cytoplasmic lipid droplets (LDs) [59]. *RNF213* oligomerizes on LD surfaces, enhancing their stability by preventing lipolysis through ATPase- and E3 ligase-dependent displacement of adipose triglyceride lipase [59,60,61]. Disease-associated mutations in the RING domain impair this function, linking lipid dysregulation to moyamoya pathogenesis. In vascular cells, impaired lipid metabolism may alter membrane composition, oxidative stress responses, and inflammatory signaling, contributing to endothelial dysfunction. *RNF213* deficiency has been shown to increase free fatty acid-induced oxidative stress and inflammatory signaling in endothelial and smooth muscle cells [55,56,57].

### 3.6. Angiogenic Dysregulation

Zebrafish (*Danio rerio*), with transparent vasculature and conserved angiogenic signaling pathways, provide an effective in vivo system for examining *RNF213* function. *RNF213* knockout (KO) zebrafish display abnormal cranial and ocular vasculature, including excessive vascular branching and defective pruning of retinal vessels—phenotypes that recapitulate angiogenic dysregulation observed in *RNF213*-deficient human endothelial cells (ECs) [6,41]. TALEN-induced *RNF213* mutations further demonstrate hyperplastic but disorganized vessel growth, resembling the pathological neovascularization characteristic of MMD [62].

### 3.7. Stress-Dependent Vascular Vulnerability

Constitutive *RNF213* KO mice are viable and develop normally without spontaneous intracranial stenosis or moyamoya-like vessels under physiological conditions [44,63]. Histological analysis confirms preserved arterial architecture, consistent with the incomplete clinical penetrance seen in human p.R4810K carriers. However, under hemodynamic stress, KO mice exhibit impaired vascular responses. For example, in chronic cerebral hypoperfusion models such as bilateral carotid artery stenosis (BCAS), *RNF213* KO mice show lower cerebral blood flow, increased infarct volume, and reduced collateral formation [5,63]. Similarly, after unilateral carotid ligation, KO mice fail to develop compensatory intimal thickening [46]. These findings suggest that *RNF213* is not essential for maintaining baseline vascular integrity but is critical for adaptive remodeling in response to stress.

### 3.8. Endothelial Specificity

To explore gain-of-function effects, transgenic mice overexpressing wild-type or p.R4810K mutant human *RNF213* have been developed. Endothelial-specific expression of the p.R4810K variant (murine R4757K) leads to impaired angiogenic responses following hypoxia or BCAS, including reduced capillary density and delayed reperfusion. In contrast, smooth muscle-specific overexpression has no such effect, highlighting the importance of endothelial cells as the primary site of *RNF213* activity [44,64]. Notably, these transgenic models do not develop spontaneous MMD-like vasculopathy, even in aged animals, reinforcing the need for additional environmental or genetic “second hits” to initiate disease. Knock-in mice carrying the human p.R4810K variant similarly do not develop moyamoya-like lesions at baseline or after carotid ligation, further supporting the requirement of cofactors beyond the mutation itself for disease manifestation [44].

### 3.9. Functional Impairments in iPSC-Derived Vascular Cells Harboring RNF213 Mutations

Induced pluripotent stem cell (iPSC) models have enabled mechanistic studies of *RNF213* mutations in human vascular cells. Endothelial cells derived from iPSCs of MMD patients (*RNF213* p.R4810K carriers) consistently exhibit diminished angiogenic potential, impaired migration, and downregulation of extracellular matrix components crucial for vessel stabilization [65,66]. iPSC-derived vascular smooth muscle cells (VSMCs) also show proliferative dysregulation and phenotypic switching, with Wnt pathway activation paralleling the intimal hyperplasia seen in MMD histology. These findings indicate that while endothelial dysfunction is likely primary, VSMC abnormalities may contribute to disease progression.

### 3.10. Pathological Significance of Rare RNF213 Variants Including Truncating Mutations

Accumulating evidence indicates that other rare *RNF213* variants—particularly truncating mutations and missense variants in the RING finger domain—also contribute to disease pathogenesis through distinct mechanisms.

Several studies have identified loss-of-function truncating mutations that abolish the E3 ubiquitin ligase activity of *RNF213*. These variants have been reported in both familial and sporadic MMD, as well as in other forms of systemic vasculopathy [1,11,39,43]. Notably, truncating mutations in the C-terminal region, especially those affecting the RING domain, disrupt the ubiquitination function of *RNF213*, impair vascular remodeling, and may lead to high-penetrance, early-onset phenotypes. Importantly, rare truncating mutations have also been documented in non-East Asian populations and in patients with diffuse occlusive vasculopathies lacking classical moyamoya angiographic features. Recent reports of de novo or compound heterozygous mutations have been associated with fulminant juvenile-onset phenotypes and syndromic presentations involving systemic vascular involvement [27,40]. These include cases with early-onset pulmonary hypertension, aortic arch anomalies, and generalized arteriopathy.

Collectively, these findings underscore the pathogenic relevance of *RNF213* variants beyond p.R4810K and highlight the importance of both mutation type (e.g., missense vs. truncating) and location (e.g., RING domain vs. ATPase domain) in determining clinical severity. Variant-specific pathogenic mechanisms—ranging from dominant-negative effects to loss of function—may explain the phenotypic diversity observed in RRV. This supports a broader conceptual framework in which RRV is not solely limited to p.R4810K but includes a spectrum of mutation-driven arteriopathies that may differ in penetrance, severity, and systemic involvement.

### 3.11. Summary

*RNF213* is a multifunctional protein that integrates angiogenic regulation, endothelial barrier maintenance, inflammatory signaling, and metabolic adaptation. The p.R4810K variant disrupts these processes, predisposing carriers to impaired collateral formation and hemodynamic vulnerability. While much evidence points toward a hypermorphic or dominant-negative mechanism, the precise molecular impact of p.R4810K remains under investigation. Continued research into *RNF213*’s ATPase, ubiquitin ligase, and oligomerization activities will be crucial for unraveling its role in cerebrovascular diseases, such as MMD and RRV, and for identifying potential therapeutic targets [41,48,49,59].

## 4. Cerebrovascular Manifestations

### 4.1. Arterial Remodeling 

RRV presents clinically as a spectrum of intracranial large-vessel diseases, ranging from asymptomatic arterial narrowing to IS attributed to ICASO or full-blown MMD [1,16]. A distinguishing feature of RRV, compared to conventional atherosclerosis, is its characteristic arterial remodeling. In *RNF213* variant carriers, intracranial stenoses typically show negative remodeling, marked by concentric narrowing with minimal outward compensatory enlargement [67,68,69]. This mirrors the vascular pathology of MMD, where smooth muscle proliferation and intimal thickening reduce luminal diameter without eccentric plaque formation. In contrast, atherosclerosis is associated with positive remodeling, featuring eccentric plaques and vessel wall expansion. High-resolution MRI studies corroborate these differences: *RNF213*-associated stenoses tend to exhibit thin vessel walls and concentric narrowing, while atherosclerotic lesions often show eccentric wall thickening and outward remodeling [1,16].

### 4.2. Risk Factor Profile

One notable clinical feature of *RNF213* p.R4810K carriers is a reduced burden of conventional cardiovascular risk factors relative to non-carriers with similar intracranial stenoses. In a multicenter study of 112 Japanese patients with ICASO (excluding overt MMD), Ohara et al. [70] found that *RNF213* carriers were significantly younger and had lower Framingham Cardiovascular Risk Score [71], Essen Stroke Risk Score [72], and Suita risk scores [73] (e.g., mean Framingham score: 10.7 vs. 15.3; *p* = 0.001). Although individual risk factors (e.g., hypertension, diabetes, smoking) were not significantly different between groups, their cumulative burden was consistently lower among carriers. These findings suggest a non-atherosclerotic pathogenesis of *RNF213*-related stenoses, congruent with MMD-like mechanisms [41]. Epidemiologically, RRV predominantly affects younger female individuals in East Asian populations where the p.R4810K variant is more prevalent. Another clinical clue is thyroid autoimmunity. Elevated anti-thyroid peroxidase antibodies (TPO-Ab), indicative of Hashimoto’s thyroiditis, are frequently observed in patients with *RNF213*-related ICASO and MMD. In a large study, 27% of *RNF213* carriers with IS or TIA had elevated TPO-Ab, compared to only 4% of non-carriers, corresponding to an adjusted OR of ~12 [74]. Though causal mechanisms are unclear, chronic autoimmune inflammation may contribute to endothelial dysfunction or negative remodeling, acting as a “second hit” in genetically susceptible individuals [14,74]. This highlights the need to evaluate thyroid function and autoimmunity in suspected RRV cases.

Moreover, thyroid dysfunction may further exacerbate vascular risk through its effects on lipid metabolism. For instance, thyroid hormone dysregulation impacts lipid metabolism by reducing low-density lipoprotein receptor activity and altering bile acid synthesis, which promotes hyperlipidemia [75,76]. These lipid abnormalities aggravate oxidative stress and endothelial dysfunction [77,78], processes that may potentiate *RNF213*-mediated vascular injury. Accordingly, the convergence of thyroid dysfunction and lipid dysregulation could serve as a mechanistic “second hit,” heightening vascular susceptibility and disease progression in RRV.

### 4.3. Natural History and Outcomes

RRV is a progressive arteriopathy, even when initially presenting with mild symptoms. A large cohort study of 753 patients with ICASO found that *RNF213* mutation carriers experienced significantly higher rates of recurrent cerebrovascular events. Recurrent stroke occurred in 17.0% of carriers versus 8.2% of non-carriers (*p* < 0.01), while TIAs (10.7% vs. 6.5%) and hemorrhagic strokes (1.7% vs. 0.2%) were also more frequent among carriers [17]. Moreover, 9.3% of *RNF213* variant carriers developed MMD-like angiographic changes, compared to only 1.3% of non-carriers, indicating a potential progression from ICASO to MMD. These data underscore the dynamic nature of RRV. Progressive vessel narrowing and the formation of fragile moyamoya collaterals increase the long-term risk of ischemic and hemorrhagic events. Although patients with IS are the most common presentation, intracerebral hemorrhage is a recognized complication, particularly in east Asian populations, where 20–40% of MMD cases present with hemorrhage. Whether *RNF213* carriers without angiographic MMD have an inherently higher hemorrhagic risk remains unclear, but immature collateral formation likely contributes to vulnerability in advanced RRV [17].

### 4.4. Genotype–Phenotype Correlation of RNF213 p.R4810K Variant in Cerebrovascular Disease

Emerging evidence indicates a clear genotype–phenotype correlation associated with the *RNF213* p.R4810K variant. Several clinical and genetic studies have demonstrated that individuals carrying the homozygous p.R4810K mutation generally present with an earlier onset of disease, more extensive and rapidly progressive intracranial steno-occlusive lesions, and a higher frequency of bilateral involvement compared to heterozygotes [3,11,27]. Homozygous carriers are also more likely to develop severe phenotypes, including early-onset MMD in childhood, recurrent ischemic or hemorrhagic strokes, and resistance to standard therapies [28,71,72,73]. Recent genetic studies have elucidated a clear genotype–phenotype correlation associated with the *RNF213* p.R4810K variant in MMD. In a large Japanese cohort, Miyatake et al. demonstrated that patients homozygous for the c.14576G>A (p.R4810K) variant experience disease onset at a much younger age—typically in early childhood—with a median age of onset of 3 years, compared to 7 years for heterozygotes and 8 years for wild-type individuals. Notably, all homozygous patients presented with IS as their initial manifestation and exhibited a higher prevalence of bilateral and severe intracranial arterial lesions [71]. Furthermore, an illustrative sibling case confirmed this genotype–phenotype relationship: while the homozygous child developed rapidly progressive, widespread MMD in infancy, the heterozygous sibling manifested much later in adulthood, displaying a considerably milder phenotype. These findings consistently indicate that homozygosity for the *RNF213* p.R4810K variant confers a markedly more severe and early-onset form of MMD, while heterozygosity is generally associated with later onset and milder clinical course [72]. Collectively, these results provide robust evidence for a gene dosage effect, whereby the number of mutant *RNF213* alleles directly influences disease severity and clinical presentation. In contrast, heterozygous carriers tend to exhibit later onset, milder or even asymptomatic forms of the disease, and occasionally present with isolated large-artery atherosclerotic stroke or non-moyamoya ICASO. These findings support the idea that the gene dosage of mutant *RNF213* alleles directly influences disease severity and clinical course [6,16,21].

### 4.5. Advanced Neuroimaging Features in RNF213 Variant Carriers

Recent advances in neuroimaging have delineated distinct vascular features in individuals harboring *RNF213* variants. High-resolution vessel wall MRI commonly reveals concentric, non-eccentric stenotic lesions with negative remodeling and thinned vessel walls [68,79]—features that stand in contrast to the eccentric plaques and positive remodeling characteristic of atherosclerosis. Furthermore, *RNF213* variant carriers frequently exhibit anatomical variants of the circle of Willis, such as hypoplastic anterior communicating arteries or bilateral posterior communicating arteries, implicating genetic influences on cerebrovascular development [80,81].

## 5. Therapeutic Perspectives

The recognition of RRV as a distinct clinical entity carries important implications for the development of tailored therapeutic and preventive strategies. Currently, no pharmacologic interventions are known to reverse the underlying arteriopathy in RRV or MMD. Surgical revascularization remains the primary treatment for symptomatic MMD, aiming to enhance cerebral perfusion through direct or indirect bypass procedures. However, recent advances in understanding the molecular biology of *RNF213* have opened avenues for genome-informed therapeutic approaches [54].

### 5.1. Modifying Second-Hit Triggers

One promising avenue involves targeting modifiable “second-hit” factors that may accelerate disease progression in genetically susceptible individuals. Among these, autoimmune thyroid disease—especially the presence of elevated anti-TPO antibodies—has been recurrently associated with RRV. Although causality has not been definitively established, maintaining euthyroid status and treating overt thyroid dysfunction (e.g., Hashimoto’s thyroiditis) may mitigate endothelial inflammation. If future studies confirm a pathogenic role for anti-TPO antibodies, immunomodulatory interventions—such as B-cell depletion therapies—could be considered in select patients with progressive arteriopathy and serological evidence of thyroid autoimmunity. More broadly, managing pro-inflammatory states may offer benefit, given the regulatory role of *RNF213* in interferon-related and inflammatory signaling pathways [1,4,50,82,83,84,85,86]. Strategies such as aggressive infection control, minimizing chronic inflammatory conditions, and reducing oxidative stress may help preserve vascular integrity in *RNF213* variant carriers.

Moreover, potential second hits include inflammatory triggers (e.g., infection, interferon elevation), hypoxia, oxidative stress, immune dysregulation, and mitochondrial dysfunction [6,87,88]. Differences in vascular wall structure, endothelial shear stress, or exposure to environmental factors like smoking or air pollution may further unmask the latent phenotype. Understanding and modifying these cofactors may be critical for disease prevention in carriers.

### 5.2. Promoting Angiogenesis and Collateral Formation

Given the anti-angiogenic phenotype associated with the *RNF213* protein harboring the p.R4810K variant, pharmacologic promotion of collateral vessel development represents another potential therapeutic strategy. Although robust clinical data are lacking, several pharmacologic agents have shown promise:

### 5.3. Medications

Antiplatelet Agents: Aspirin remains standard for secondary stroke prevention in patients with ICASO and MMD. While not specific to *RNF213*, it may reduce microthrombosis and endothelial dysfunction.

Cilostazol: Cilostazol, a phosphodiesterase III inhibitor with vasodilatory and proangiogenic effects, has been used empirically in MMD to enhance perfusion. A small pilot study suggested that switching from cilostazol to its metabolite OPC-13015 led to improved cognitive function in patients carrying *RNF213* variants, hinting at possible genotype-guided benefits [44].

Statins: Beyond lipid-lowering, statins enhance nitric oxide bioavailability and angiogenesis. While clinical evidence remains anecdotal, case reports suggest potential utility in promoting collateral vessel development in MMD. Given their favorable safety profile and pleiotropic vascular effects, statins merit further investigation as adjunctive therapy in RRV. Supporting this, a 15-year follow-up study involving patients with intracranial artery stenosis demonstrated that statin use significantly reduced the risk of stenosis progression in *RNF213* p.R4810K variant carriers (hazard ratio 0.20, 95% CI 0.06–0.63, *p* = 0.006), but not in non-carriers, indicating a potential genotype-specific therapeutic effect. These findings provide real-world support for the role of statins as a vascular stabilizer in the setting of RRV [89].

### 5.4. Intervention

In patients with *RNF213*-related PAH, balloon pulmonary angioplasty (BPA) has demonstrated promise for improving hemodynamic parameters and reducing symptom burden. Although the clinical evidence is still limited, several case reports have documented favorable outcomes with BPA in genetically confirmed *RNF213* variant carriers [90]. Similarly, in systemic arteries such as the renal or iliac vessels, endovascular stenting may be considered in cases of critical stenosis; however, the risk of restenosis remains a significant concern. These emerging interventional approaches highlight the potential of genotype-informed vascular therapies in select patient subsets. Nevertheless, the broader therapeutic landscape for RRV is still in its infancy. A comprehensive precision medicine framework—integrating genetic status, vascular phenotype, and immune profile—may ultimately guide individualized management strategies. In addition to interventional options, pharmacologic and immunomodulatory therapies targeting angiogenesis, inflammation, and endothelial function warrant rigorous evaluation in genetically defined populations. In a multicenter cohort of patients with anterior-circulation large-vessel occlusion stroke undergoing endovascular therapy (EVT), carriers of the *RNF213* p.R4810K variant exhibited significantly higher rates of instant re-occlusion during the procedure (70.0% vs. 5.6%) and early re-occlusion within two weeks (60.0% vs. 0.4%) compared with non-carriers. Despite these procedural complications, final reperfusion success—as defined by a modified thrombolysis in cerebral infarction grade ≥2b—was achieved in 100.0% of variant carriers versus 81.6% of non-carriers [91]. These findings suggest that rapid *RNF213* genotyping may enable genotype-guided device selection, adjunctive therapy planning, and post-procedural surveillance, particularly in acute stroke care settings. These considerations underscore the need for future clinical trials to evaluate both medical and interventional approaches in the context of RRV.

Candidate pharmacologic therapies and mechanisms in RRV are shown in Table 1.

## 6. Conclusions

RRV represents a genetically determined, non-atherosclerotic arteriopathy that challenges traditional classifications of intracranial large-vessel disease. The discovery of the *RNF213* p.R4810K variant has not only expanded our understanding of MMD but also revealed a broader disease spectrum, including isolated ICASO, recurrent IS, and subclinical vasculopathy. Importantly, a growing body of clinical evidence indicates that carriers of this variant—particularly in east Asian populations—exhibit distinctive features such as younger age of onset, absence or low burden of conventional cardiovascular risk factors, female predominance, and coexisting autoimmune traits, such as thyroid autoimmunity. These characteristics may serve as important clinical clues to identify individuals who are likely to harbor the *RNF213* variant. Recognizing these phenotypic patterns is essential for differentiating RRV from atherosclerotic cerebrovascular disease and for considering early genetic testing. Emerging data from molecular studies, animal models, and patient-derived iPSC systems further underscore *RNF213*’s central role in vascular homeostasis, endothelial signaling, and metabolic regulation. Despite these advances, critical questions remain regarding the precise pathophysiological mechanisms, environmental or immunologic “second hits,” and optimal strategies for personalized risk stratification and therapeutic intervention.

Clinically, early identification of *RNF213* variant carriers based on their characteristic presentation may enable more precise diagnosis and individualized management. Future work should aim to refine diagnostic criteria, establish genotype-guided treatment algorithms, and explore potential therapeutic targets modulating *RNF213* function or its downstream pathways. Understanding RRV as a unified and genetically heterogeneous disease concept can bridge the gap between MMD and non-MMD ICASO, ultimately improving clinical outcomes through tailored interventions.

## Figures and Tables

**Table 1:** Candidate pharmacologic therapies and mechanisms in *RNF213*-related vasculopathy.

Therapeutic Class	Candidate(s)	Proposed Mechanism	Supporting Evidence	Current Status/Challenges
Statins	Atorvastatin Rosuvastatin	↑ eNOS activity ↓ Oxidative stress ↑ NO bioavailability	HR 0.20 for stenosis progression in *RNF213* carriers	Observational data only; trials needed
Angiogenic Agents	VEGF, HGF, FGF2	Promotion of collateral vessel development	Preclinical models; case reports in MMD suggest potential benefit	Primarily preclinical; limited trials
Immunomodulatory Drugs	Rituximab Corticosteroids	Suppression of vascular inflammation	High prevalence of autoimmune thyroiditis in *RNF213* carriers suggests a potential pathogenic role	Hypothesis-driven; untested clinically

Abbreviation: eNOS, endothelial Nitric Oxide Synthase; FGF2, Fibroblast Growth Factor 2, HGF, Hepatocyte Growth Factor; HR, Hazard Ratio; MMD, Moyamoya Disease; NO, Nitric Oxide; VEGF, Vascular Endothelial Growth Factor. Arrows indicate direction of effect: ↑ denotes increase or stimulation, and ↓ denotes decrease or inhibition.
